# Distinct expression and localization of the type II diacylglycerol kinase isozymes δ, η and κ in the mouse reproductive organs

**DOI:** 10.1186/s12861-015-0055-z

**Published:** 2015-01-23

**Authors:** Takao Shionoya, Takako Usuki, Suguru Komenoi, Takeshi Isozaki, Hiromichi Sakai, Fumio Sakane

**Affiliations:** Department of Chemistry, Graduate School of Science, Chiba University, 1–33 Yayoi-cho, Inage-ku, Chiba 263-8522 Japan

**Keywords:** Diacylglycerol kinase, Alternative splicing, Spermatocyte, Ovarian follicle, Uterine luminal epithelium

## Abstract

**Background:**

We have revealed that the type II diacylglycerol kinases (DGKs) δ, η and κ were expressed in the testis and ovary. However, these enzymes’ functions in the reproductive organs remain unknown.

**Results:**

In this study, we first identified the expression sites of type II DGKs in the mouse reproductive organs in detail. Reverse transcription-polymerase chain reaction and Western blotting confirmed that DGKδ2 (splicing variant 2) but not DGKδ1 (splicing variant 1) and DGKκ were expressed in the testis, ovary and uterus. DGKη1 (splicing variant 1) but not DGKη2 (splicing variant 2) was strongly detected in the ovary and uterus. Interestingly, we found that a new alternative splicing product of the DGKη gene, DGKη3, which lacks exon 26 encoding 31 amino acid residues, was expressed only in the testis. Moreover, we investigated the distribution of type II DGKs in the testis, ovary and uterus through *in situ* hybridization. DGKδ2 was distributed in the primary spermatocytes of the testis and ovarian follicles. DGKη1 was distributed in the oviductal epithelium of the ovary and the luminal epithelium of the uterus. Intriguingly, DGKη3 was strongly expressed in the secondary spermatocytes and round spermatids of the testis. DGKκ was distributed in the primary and secondary spermatocyte of the testis.

**Conclusion:**

These results indicate that the expression patterns of the type II DGK isoforms δ2, η1, η3 and κ differ from each other, suggesting that these DGK isoforms play specific roles in distinct compartments and developmental stages of the reproductive organs, especially in the processes of spermatogenesis and oocyte maturation.

## Background

Diacylglycerol kinase (DGK) phosphorylates diacylglycerol (DG) to generate phosphatidic acid (PA) [1-4]. DG, which is liberated from phosphatidylinositol 4,5-bisphosphste and phosphatidylcholine upon cell stimulation, regulates a wide range of cellular functions. For instance, DG is an allosteric activator of conventional and novel protein kinase Cs (PKCs), Unc-13 and Ras guanyl nucleotide-releasing protein [5-7]. Therefore, DGK consumes DG and is thus responsible for attenuating DG-mediated signals. PA, the reaction product of DGK, has also been reported to regulate a number of signaling proteins such as phosphatidylinositol-4-phosphate 5-kinase, Ras GTPase-activating protein, C-Raf, mammalian target of rapamycin and atypical PKC [8-10]. Therefore, DGK is thought to play roles not only in the downregulation of DG signaling but also in the production of another lipid mediator, PA.

It is well known that DGK represents a large enzyme family. Ten mammalian DGK isozymes, namely α, β, γ, δ, ε, ζ, η, θ, ι, and κ, which contain two or three characteristic cysteine-rich, zinc finger-like C1 domains and the catalytic region in common, are subdivided into five groups according to their structural features [1-4]. The type II DGK [11] comprises the δ [12], η [13] and κ [14] isozymes. The occurrence of alternative splicing was reported for DGKδ (δ1 and δ2) [15] and DGKη (η1 and η2) [16]. All of the type II DGK isoforms possess a pleckstrin homology domain at their N termini and a separated catalytic domain, and DGKs δ1, δ2 and η2 but not DGKs η1 and κ contain a sterile α-motif domain at their C termini. It has been reported that DGKs δ1, δ2 and η2 formed oligomers through interactions among their sterile α-motif domains and that this oligomer formation regulates the activities and subcellular localizations of these DGK isoforms [15-19]. DGKs δ2 and κ contain the Pro-rich 52 residues [15] and the 33 tandem repeats of Glu-Pro-Ala-Pro [14] extending from the N terminus, respectively.

Based on the analysis of DGKδ-knockout mice, it was recently reported that DGKδ regulates the epidermal growth factor receptor pathway in epithelial cells of the lung and skin [20] and insulin receptor signaling in skeletal muscle [21,22] by modulating PKC activity. We recently reported that DGKη is expressed in stomach cancer and HeLa cervical cancer cells and that it is required for the Ras/B-Raf/C-Raf/MEK/ERK signaling cascade, which is activated by the epidermal growth factor [23]. Type II DGKs have been implicated in several diseases [24]. For example, DGKδ is a key enzyme that prevents insulin resistance and type 2 diabetes [21]. A female patient with a disrupted DGKδ gene who exhibits seizures and a psychiatric disorder was found [25]. DGKη was reported to be involved in lung cancer [26]. A genome-wide association study implicated the DGKη gene in the etiology of bipolar disorder [27,28]. A genome-wide association study also indicated a potential relationship between DGKκ and hypospadias [29].

Several reports have revealed that DGKδ, η and κ are abundantly expressed in the reproductive organs, testis and ovary [12-16]. However, the functions of DGKs δ, η and κ in the reproductive organs remain unclear, and not even their detailed distribution patterns in the organs have been revealed. Therefore, in this study, we examined the expression and spatial distribution of the DGKs δ2, η1 and κ proteins and mRNAs in the mouse reproductive organs. The results indicate distinct expression patterns, which were obviously different from each other. Moreover, we found a new splice variant of DGKη, DGKη3, which was specifically expressed in the testis.

## Results

### Expression of DGKδ, η and κ in the mouse reproductive organs

**Figure 1:**
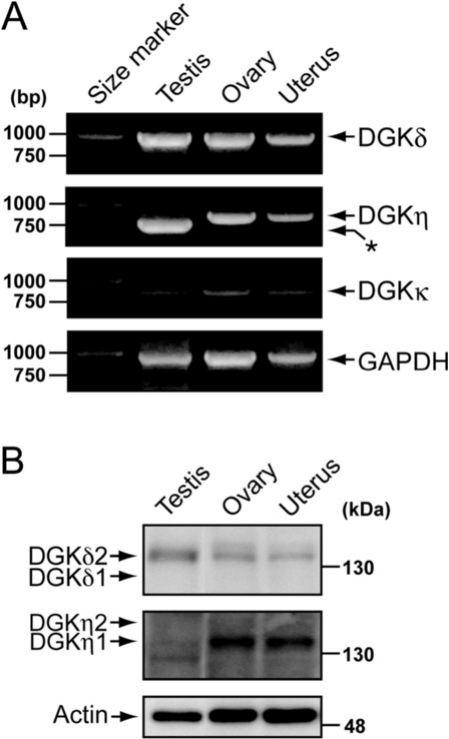
**Expression of type II DGK mRNAs (A) and proteins (B) in the reproductive organs: testis, ovary and uterus. (A)** RT-PCR analysis of the mRNA from the reproductive organs was performed: testes from 12-week-old male mice and ovaries and uteruses from 12-week-old female mice. The panels show that 999-bp cDNA fragments were amplified for DGKδ (35 cycles), 826-bp cDNA fragments were amplified for DGKη (35 cycles), 842-bp cDNA fragments were amplified for DGKκ (35 cycles) and 978-bp cDNA fragments were amplified for mouse glyceraldehyde phosphate dehydrogenase (GAPDH, 35 cycles), as determined through agarose gel electrophoresis. The asterisk indicates a ~730-bp band that was amplified from the testis mRNA. Representatives of five independent experiments with five male and five female mice are shown. **(B)** The protein samples (30 μg) from the indicated tissues of 10-week-old male and female mice were detected by Western blotting using anti-DGKδ and DGKη antibodies. Representatives of five independent experiments with five male and five female mice are shown.

We first confirmed the expression of DGKδ mRNA and protein in the mouse reproductive organs: the testis, ovary and uterus. The 999-bp cDNA fragment amplified from DGKδ mRNA was strongly detected in the testis and ovary and was clearly observed in the uterus by reverse transcription-polymerase chain reaction (RT-PCR) (Figure 1A). The Western blot analysis showed that the DGKδ2 protein (the splice variant 2, calculated molecular mass: 135 kDa [15]) was substantially expressed in the testis and ovary and, to a lesser extent, in the uterus (Figure 1B). The DGKδ1 protein (the splice variant 1, calculated molecular mass: 130 kDa [12]) was not detectable in the reproductive organs (Figure 1B).

The RT-PCR results demonstrated that DGKη mRNA was expressed strongly in the testis and ovary and moderately expressed in the uterus (Figure 1A). The RT-PCR product using DGKη mRNA from the testis (approximately 730 bp) was shorter than those obtained from the ovary and uterus (the 826-bp cDNA fragment) (Figure 1A). Therefore, the shorter product (~730 bp) is specifically generated in the testis. The Western blot analysis showed that the DGKη1 protein (the splice variant 1, calculated molecular mass: 128 kDa [13]) was strongly detected in the ovary and uterus (Figure 1B). The DGKη1 protein was detected at a slightly higher position (132 kDa) in the SDS-polyacrylamide gel electrophoresis results than that corresponding to the calculated molecular mass (Figure 1B). The DGKη2 protein (the splice variant 2, calculated molecular mass: 135 kDa (139 kDa in SDS-polyacrylamide gel electrophoresis) [16]) was not detected in the reproductive organs.

**Figure 2:**
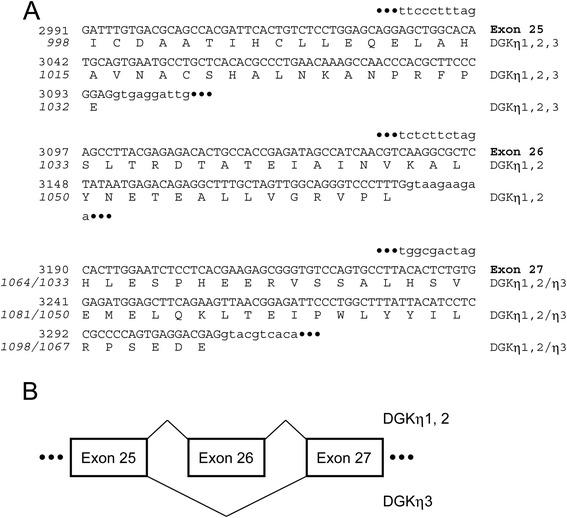
**Alternative splicing products of DGKη. (A)** The nucleotide sequences and deduced amino acid sequences of DGKη1, DGKη2 and DGKη3 are shown. The nucleotide and amino acid numbers are indicated in the left in italic and plain text, respectively. **(B)** The exon-intron structure of the DGKη gene (exons 25–27) and the alternative splicing that yields the two different forms (DGKη1/2 and DGKη3) are shown.

The sequence analysis of the RT-PCR product amplified from the testis mRNA revealed that the shorter mRNA (precisely 733 bp) is an alternative splicing product of the DGKη gene in which exon 26 is skipped (Figure 2). Therefore, we designate this product DGKη3. Exon 26 of the DGKη gene contains 93 bp and encodes 31 amino acid residues. Therefore, no frame shift occurs due to alternative splicing. The exon/intron boundaries between exons 25 and 27 fulfill the GT/AG rule (Figure 2A). In addition to the ovary and uterus, the 733-bp band failed to be detected in the epididymis, sperm, vesicula seminalis, prostate gland, brain and Neuro 2a neuroblastoma cells (data not shown). These results further strongly suggest that DGKη3 is specifically expressed in the testis. The alternative splicing product is anticipated to produce a shorter DGKη protein with an expected molecular mass of 125 kDa (129 kDa as detected by SDS-polyacrylamide gel electrophoresis). We detected a 129-kDa band that reacted with anti-DGKη antibody in the testis (Figure 1B). However, we found that this band was also detected (data not shown) in the extracts from the testis of a recently created DGKη-knockout mouse (Isozaki, T. *et al.*, unpublished work). Therefore, although DGKη3 mRNA was strongly expressed in the testis (Figure 1A), its protein band has not yet been identified (Figure 1B). The DGKη3 protein may be unstable and quickly degraded.

DGKκ mRNA was modestly expressed in the testis, ovary and uterus (Figure 1A). DGKκ protein was not detected in these mouse reproductive organs by Western blotting using anti-human DGKκ antibody (data not shown). Anti-DGKκ antibody against human DGKκ [14] may not effectively recognize the murine DGKκ protein.

### Distribution of DGKδ, η and κ mRNAs in the testis

**Figure 3:**
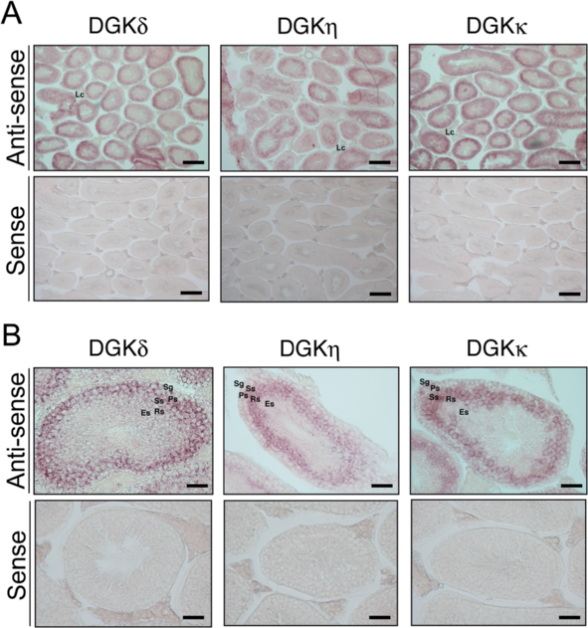
***In situ*** **hybridization of the type II DGK mRNAs in the testis of a 12-week-old male mouse. (A)** The type II DGK mRNAs were hybridized and detected with the antisense probe of the type II DGK mRNAs. The testis sections were also hybridized with the sense probes as controls. **(B)** High-magnification image of the seminiferous tubules. Representatives of three independent experiments with three male mice are shown. Sg, spermatogonium; Ps, primary spermatocyte; Ss, secondary spermatocyte; Rs, round spermatid; Es, elongated spermatid; Lc, Leydig cell. The scale bars in **(A)** represent 200 μm, and the scale bars in **b** represent 40 μm.

We then performed *in situ* hybridization using the anti-sense probe to examine the spatial distribution of DGKδ2 mRNA in the mouse testis (Figure 3). Although the testis sections were also hybridized with the sense probe as a control, no distinct staining was detected (Figure 3). As shown in Figure 3, DGKδ mRNA was mainly expressed in primary spermatocytes. The mRNA was moderately detected in the spermatogonia and, to a lesser extent, in the secondary spermatocytes. However, no obvious staining of DGKδ mRNA was detected in the round and elongated spermatids and the Leydig cells. The expression intensities of DGKδ in these testicular cells are summarized in Table 1.

**Table 1:** **Expression of type II DGKs in the testis**

**Testis**						
	**Spermatogonium**	**Primary spermatocyte**	**Secondary spermatocyte**	**Round spermatid**	**Elongated spermatid**	**Leydig cell**
DGK δ	++	+++	+	–	–	–
DGK η	–	+	++	+++	–	–
DGK κ	+	++	+++	+	–	–

– : not detected; + : weakly detected; ++: moderately detected; +++: strongly detected.

The RT-PCR results demonstrated that DGKη3 mRNA but not DGKη1/2 mRNA was mainly detected in the testis (Figure 1). Compared with DGKδ2 mRNA, DGKη3 mRNA was detected in the inner area of the testis. DGKη3 mRNA was strongly expressed in the secondary spermatocytes and the round spermatids and was weakly detected in the primary spermatocytes (Figure 3 and Table 1). In the spermatogonia, elongated spermatids and the Leydig cells, DGKη3 mRNA was not detectable. No distinct staining was detected with the sense probe as a control (Figure 3).

Compared with the DGKδ and DGKη mRNAs, DGKκ mRNA was expressed in the intermediate region between these isozymes in the testis. DGKκ mRNA was substantially detected in the primary and secondary spermatocytes and weakly distributed in the spermatogonia and round spermatids (Figure 3 and Table 1). However, no obvious hybridization with DGKκ mRNA was detected in the round and elongated spermatids and the Leydig cells. No detectable staining was observed with the sense probe as a control (Figure 3).

### Distribution of DGKδ, η and κ mRNAs in the ovary and oviduct

**Figure 4:**
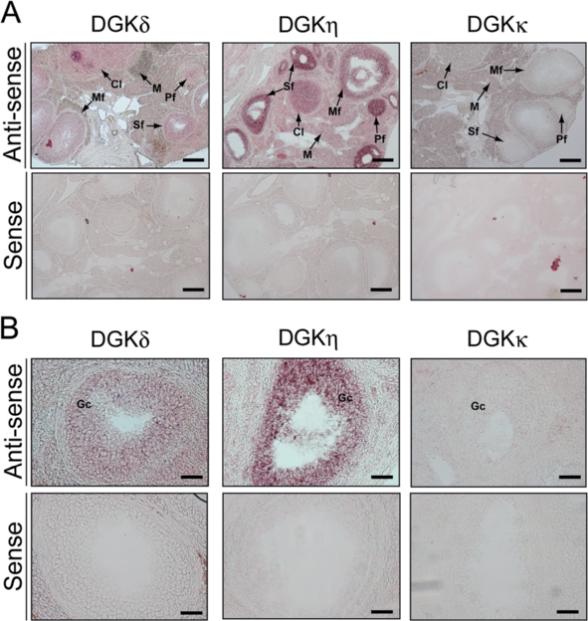
***In situ*** **hybridization of the type II DGK mRNAs in the ovary of a 12-week-old female mouse. (A)** The type II DGK mRNAs were hybridized and detected with the antisense probes of the type II DGK mRNAs. The ovary sections were also hybridized with the sense probes as controls. **(B)** High-magnification image of the ovarian follicles (secondary follicles). Although we observed histological specimens from five female mice in different estrous cycles, essentially the same results were obtained. Representatives of five independent experiments with five female mice are shown. Pf, primary follicle; Sf, secondary follicle; Mf, mature follicle; Cl, corpus luteum; Gc, granulosa cell; M, medulla. The scale bars in **(A)** represent 200 μm, and the scale bars in **(B)** represent 40 μm.

**Figure 5:**
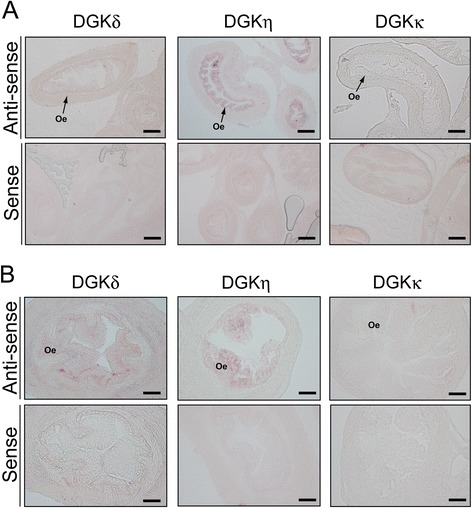
***In situ*** **hybridization of the type II DGK mRNAs in the oviduct of a 12-week-old female mouse. (A)** The type II DGK mRNAs were hybridized and detected with the antisense probes of the type II DGK mRNAs. The oviduct sections were also hybridized with the sense probes as controls. **(B)** High-magnification image of the ovarian oviduct. Representatives of four independent experiments with four female mice are shown. Oe, oviductal epitherium. The scale bars in **(A)** represent 100 μm, and the scale bars in **(B)** represent 40 μm.

DGKδ mRNA was broadly and modestly expressed in the primary, secondary and mature follicles and the corpus lutea (Figure 4 and Table 2). In the medulla, weak staining was observed. DGKδ mRNA was weakly detected in the oviductal epithelium (Figure 5). Although the ovary and oviduct sections were also hybridized with the sense probe as a control, no distinct staining was detected (Figures 4 and 5).

**Table 2:** **Expression of type II DGKs in the ovary and oviduct**

**Ovary**						
	**Primary follicle**	**Secondary follicle**	**Mature follicle**	**Corpus luteum**	**Medulla**	**Oviductal epithelium**
DGKδ	++	++	++	++	+	+
DGKη	++++	++++	++	++	+	++
DGKκ	–	–	–	–	+	–

– : not detected; +: weakly detected; ++: strongly detected; ++++: very strongly detected.

Strong staining of DGKη mRNA was observed in the granulosa cells (follicular epithelium) of the primary and secondary follicles (Figure 4 and Table 2). Moreover, the mature follicles and corpus lutea, which are matured from secondary follicles, only modestly expressed DGKη mRNA. In the medulla, only weak staining was observed. DGKη mRNA was strongly expressed in the oviductal epithelium (Figure 5 and Table 2). With the sense probe as a control, no distinct staining was detected (Figures 4 and 5).

DGKκ mRNA was slightly expressed in the medulla of the ovary (Figure 4 and Table 2). However, DGKκ mRNA failed to be detected in the ovarian follicles and corpus lutea. This mRNA was not detectable in the oviductal epitherium (Figure 5 and Table 2). No obvious staining was detected with the sense probe as a control (Figures 4 and 5).

### Distribution of DGKδ, η and κ mRNAs in the uterus

**Figure 6:**
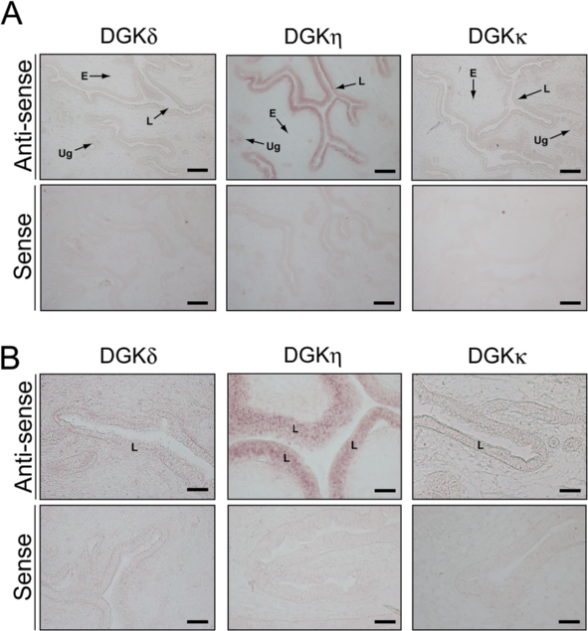
***In situ*** **hybridization of the type II DGK mRNAs in the uterus of a 12-week-old female mouse. (A)** The type II DGK mRNAs were hybridized and detected with the antisense probes of the type II DGK mRNAs. The uterus sections were also hybridized with the sense probes as controls. **(B)** High-magnification image of the endometrial epithelium. Representatives of four independent experiments with four female mice are shown. L, luminal epithelium; E, endometrium; Ug, uterine gland. The scale bars in **(A)** represent 200 μm, and the scale bars in **(B)** represent 40 μm.

DGKη mRNA was strongly expressed in the luminal epithelium but not in the endometrium (Figure 6 and Table 3). DGKη mRNA was clearly detected in the uterine glands. However, the mRNA was not detectable in the endometrium or myometrium (data not shown). No distinct staining was detected with the sense probe as a control (Figure 6).

**Table 3:** **Expression of type II DGKs in the uterus**

**Uterus**				
	**Luminal epithelium**	**Endometrium**	**Uterine gland**	**Myometrium**
DGKδ	+	–	–	–
DGKη	+++	–	+	–
DGKκ	+	–	–	–

–: not detected; +: weakly detected; +++: strongly detected.

DGKδ and DGKκ were broadly distributed and only weakly detected in the luminal epithelium of the uterus (Figure 6 and Table 3).

## Discussion

This study provides the first detailed demonstration of the expression and distribution of the type II DGK isozymes DGKδ, η and κ in the mouse reproductive organs. DGKδ, η and κ were expressed in the mouse reproductive organs, namely the testis, ovary and uterus. However, their expression patterns were obviously different from each other.

Exon 26, which encodes 31 amino acid residues, is skipped in the new alternative splicing product of the DGKη gene, which is denoted DGKη3 (Figures 1 and 2). DGKη3 was strongly expressed in the secondary spermatocytes and the round spermatids and was not detected in other tissues, such as the ovary, uterus, epididymis, sperm, vesicula seminalis, prostate gland, brain and Neuro 2a neuroblastoma cells (data not shown), implying that DGKη3 is specifically expressed in the testis, particularly in the secondary spermatocytes and the round spermatids. Therefore, this isoform plays a specialized role in these testicular cells. It is interesting to investigate the functional difference that is generated by the absence of exon 26. Although a motif search was carried out, no obvious functional motifs were not found in the 31-amino acid sequence encoded by exon 26. A protein database search indicated that the 31-amino acid sequence showed high similarity (34.5% identity (79.3% similar) in 29-aa overlap) with the C-terminal region (aa 797–825) of promyelocytic leukemia protein isoform 1, which is a tumor suppressor of acute promyelocytic leukemia [30,31]. However, the function of the region of promyelocytic leukemia protein isoform 1 has not been revealed. Therefore, the function of the 31-amino acid sequence lacking in DGKη3 remains unclear at present.

In the testis, different expression patterns of type II DGKs were observed. During the process of spermatogenesis, the primary spermatocytes are developed from the spermatogonia through mitosis [32,33]. The secondary spermatocytes are then derived from the primary spermatocytes via the first meiotic division. Moreover, the round spermatids are generated from the secondary spermatocytes through the second meiotic division. DGKδ2 mRNA was mainly expressed in the spermatogonia and primary spermatocytes (Figure 3 and Table 1). Strong staining of DGKκ was detected in the primary and secondary spermatocytes (Figure 3 and Table 1). DGKη3 mRNA was strongly expressed in the secondary spermatocytes and the round spermatids (Figure 3 and Table 1). These results imply that DGKs δ2, η3 and κ are involved in mitosis and the first and second meiotic divisions, respectively. The sperm fertilization-related protein equatorin, which is involved in fusion with the oolemma, was reported to be strongly expressed in secondary spermatocytes and round spermatids and was weakly detected in the primary spermatocytes [34]. This expression pattern of equatorin is very similar to that of DGKη3 (Figure 3). Therefore, equatorin and DGKη3 may be functionally linked to each other.

A genome-wide association study indicated that DGKκ is involved in hypospadias [29]. Hypospadias is thought to relate to androgen production [35]. Androgen is mainly produced in Leydig cells [35]. However, DGKκ was not detected in Leydig cells. Moreover, the expression pattern of DGKκ was not similar to that of androgen receptor, which was mainly expressed in spermatogonia and primary spermatocytes [36]. Therefore, a relationship between the distribution pattern of DGKκ and hypospadias was not found in this study.

In the ovary, DGKη1 mRNA was strongly expressed in the granulosa cells (follicular epithelium) of the primary and secondary follicles and modestly detected in the mature follicles and corpus luteum (Figure 4). Because granulosa cells are vigorously proliferated in the primary and secondary follicles during the maturation of ovarian follicles [37], it is possible that DGKη1 is involved in the proliferation of granulosa cells. We reported that DGKη1 enhances cell proliferation through the activation of C-Raf [23]. McPhillips, F. *et al.* reported that C-Raf (Raf-1), which is expressed in granulosa cells, mediates growth factor-stimulated growth in ovarian cancer [38]. Therefore, these results allow us to speculate that DGKη1 is involved in ovarian carcinogenesis through the activation of C-Raf (Raf-1). DGKδ2 mRNA was also expressed in the granulosa cells (follicular epithelium) of the primary and secondary follicles and modestly detected in the mature follicles and corpus luteum (Figure 4). However, its expression levels were not changed during the follicle development. Therefore, the function of DGKδ2 is likely different from that of DGKη1.

DGKη1 mRNA was strongly expressed in the luminal epithelium of the uterus (Figure 6), and DGKδ2 and κ mRNAs were weakly detected in these cells. Similar to the granulosa cells in the ovary, the luminal epithelium proliferates during the estrus cycle [37]. Epidermal growth factor receptor is known to be highly expressed in the luminal epithelium [39]. Among type II DGKs, DGKη1 enhances cell proliferation downstream of epidermal growth factor receptor [23], and DGKδ2 activates epidermal growth factor receptor through inactivation of protein kinase C [20]. Therefore, these DGK isoforms may positively regulate proliferation through the receptor in the luminal epithelium.

## Conclusions

Our data demonstrate that the spatial expression patterns of DGKs δ2, η1, η3 and κ in the murine reproductive organs are different from each other. These results support the hypothesis that DGKs δ2, η1, η3 and κ can play specific roles in distinct compartments and developmental stages of the reproductive organs, especially in the processes of spermatogenesis and oocyte maturation. In addition, we revealed the existence of a new alternative splicing product of DGKη, which we denoted DGKη3. We recently established DGKη-knockout mice (Isozaki, T. *et al.* unpublished work). To analyze the reproductive organ-related phenotypes of the knockout mice, it is important to understand the expression patterns of DGKη in the reproductive organs. Studies are underway to examine the physiological functions of DGKη1 and η3 in the reproductive organs using the newly established DGKη-knockout mice.

## Methods

### Animals and tissue preparation

C57BL/6 N mice were obtained from SLC Japan Inc. (Shizuoka, Japan). Mouse tissues were removed immediately after decapitation. All of the procedures using experimental animals were approved by the Ethics Committee of Chiba University (No. 26–96) and were performed according to the guidelines for the Care and Use of Laboratory Animals of Chiba University.

### RT-PCR

The testis, ovary and uterus from 10 to 12-week-old male and female mice were homogenized in QIAzol lysis reagent (Qiagen, Venlo, Netherlands), and the total RNA was isolated with a Direct-zol RNA MiniPrep kit (Zymo Research, Irvine, CA, USA). cDNA synthesis was performed with the Transcriptor First-Strand cDNA Synthesis Kit (Roche Diagnostics, Mannheim, Germany), using 0.5 μg of total RNA and random hexamer primers. PCR amplification was performed using rTaq polymerase (Toyobo, Osaka, Japan) and the following mouse DGKδ, η and κ-specific oligonucleotide primers. The DGKδ primers were the following: forward primer (nucleotide positions 4506–4527, 5’-CGGGATCCGGAAGTGACATATGCCATGAGA-3’) and reverse primer (nucleotide positions 5484–5505, 5’-GGGGTACCTCCTTCATTCTATCCCTCTCCA-3’). The PCR conditions were as follows: 94°C for 3 min, 35 cycles of 94°C for 30 sec, 56°C for 30 sec, and 72°C for 1.5 min, and 72°C for 5 min. The DGKη primers were the following: forward primer (nucleotide positions 2417–2436, 5’-GGGAATTCCGGGAGCTACTACAGAGATC-3’) and reverse primer (nucleotide positions 3224–3243, 5’-GGGGGTCGACCTCCACAGAGTGTAAGGCAC-3’). The PCR conditions were as follows: 94°C for 3 min, 35 cycles of 94°C for 30 sec, 58°C for 30 sec, and 72°C for 2 min, and 72°C for 5 min. The DGKκ primers were the following: forward primer (nucleotide positions 3758–3778, 5’-GGGAATTCACCTTGGGCAACTACAGTGTT-3’) and reverse primer (nucleotide positions 4581–4600, 5’-GGGGGTCGACGGGTCACTTAGAGGTCGAGT-3’). The PCR conditions were as follows: 94°C for 3 min, 35 cycles of 94°C for 30 sec, 50°C for 30 sec, and 72°C for 2 min, and 72°C for 5 min. For normalization, the mouse glyceraldehyde-3-phosphate dehydrogenase (GAPDH) mRNA was simultaneously amplified (35 cycles) using the following GAPDH-specific oligonucleotide primers: forward primer (nucleotide positions 103–128, 5’-TCGGTGTGAACGGATTTGGCCGTATT-3’) and reverse primer (nucleotide positions 1056–1079, 5’-CATGTAGGCCATGAGGTCCACCAC-3’). The PCR conditions were as follows: 94°C for 3 min, 35 cycles of 94°C for 30 sec, 45°C for 30 sec, and 72°C for 1 min, and 72°C for 5 min. The amplified PCR products were separated by agarose gel electrophoresis and stained with ethidium bromide (Wako Pure Chemical, Osaka, Japan).

The RT-PCR product amplified from the DGKη mRNA in the testis was purified and then sequenced using the DGKη-specific forward and reverse primers described above. The sequencing was carried out by Eurofins Genomics (Tokyo, Japan).

### Western blotting

The testis, ovary and uterus from 10- to 12-week-old male and female mice were homogenized in lysis buffer (50 mM HEPES, pH7.2, 150 mM NaCl, and 5 mM MgCl_2_) containing complete EDTA-free protease inhibitor cocktail (Roche Diagnostics) and centrifuged at 1,000 × *g* for 5 min. The protein concentration in the supernatants was determined using a bicinchoninic acid protein assay kit (Thermo Scientific, Hudson, NH, USA). The tissue lysates (30 μg of protein) were separated on SDS-PAGE, and the separated proteins were transferred to a polyvinylidene difluoride membrane (Pall Life Sciences, Port Washington, NY, USA). The membrane was blocked with 5% skim milk and incubated with an anti-DGKδ polyclonal antibody [15] and an anti-DGKη polyclonal antibody (ProteinTech Group, Chicago, IL, USA) overnight at 4°C. The immunoreactive bands were visualized using a peroxidase-conjugated anti-rabbit IgG antibody (Jackson ImmunoResearch Laboratories, West Grove, PA, USA) and the ECL Western Blotting Detection System (GE Healthcare Bio-Sciences, Piscataway, NJ, USA).

### Preparation of plasmids and digoxigenin-labeled Riboprobes for in situ hybridization

The PCR products of DGKs from the mouse brain were subcloned into the BamHI/KpnI and EcoRI/SalI sites of the pBluescript SK (+) vector (Stratagene-Agilent Technologies, Santa Clara, CA, USA). The plasmids were cut with either KpnI (Roche Diagnostics) or BamHI for DGKδ mRNA, and with either SalI or EcoRI for DGKη and κ mRNA to produce the sense or anti-sense probes. The digoxigenin (DIG)-labeled probes were synthesized by transcribing 3 μg of template linear DNA with T3 and T7 RNA polymerase (Promega, Madison, WI, USA) according to the DIG RNA labeling kit protocol (Roche Diagnostics).

### *In situ* hybridization

The testis, ovary and uterus isolated from 10- to 12-week-old male and female mice were rapidly frozen in 100% OCT compound (Tissue-Tek, Sakura Finetek USA, Inc., Torrance, CA, USA) in a dry ice-hexane bath. The frozen tissues were sectioned at 25-μm thickness (Leica, Solms, Germany) and mounted on APS-coated slides (S8441, Matsunami, Tokyo, Japan). The sections were fixed in 4% paraformaldehyde in phosphate buffered saline (PBS) (137 mM NaCl, 2.67 mM KCl, 8.09 mM Na_2_HPO_4_・12H_2_O, and 1.47 mM KH_2_PO_4_) (Wako Pure Chemical) for 20 min. The sections were treated with proteinase K (1 μg/μl, Qiagen) for 30 min at 37°C and fixed with 4% paraformaldehyde in PBS for 5 min. The basic proteins were acetylated with 0.25% acetic anhydrite in 0.1 M triethylamine (pH 8.0), and both before and after this process, the slides were rinsed with PBS. Pre-hybridization was carried out with hybridization buffer (40% deionized formamide, 10% dextran sulfate, 1× Denhard’s solution, 4× SSC (1× SSC: 0.15 M NaCl, and 15 mM sodium citrate), 10 mM dithiothreitol, 1 mg/ml tRNA from baker’s yeast, 1 mg/ml boiled deoxyribonucleic acid from salmon sperm, and 0.25 mg/ml DIG-labeled riboprobe denatured at 65°C for 30 min) without riboprobes for 30 min at 60°C. The hybridization was performed overnight with hybridization buffer at 60°C. At the end of the incubation period, the sections were rinsed stepwise at 60°C with 4× SSC, 2× SSC in 50% deionized formamide, and 0.1× SSC and blocked through a 30 min incubation with blocking reagent solution (Roche Diagnostic). The sections were incubated for 30 min at room temperature in the presence of anti-DIG Fab fragments conjugated with alkaline phosphatase (1/5,000 dilution) in blocking reagent solution. The staining was eventually developed using nitroblue tetrazolium/5-bromo-4-chloro-3-indolyl-phosphate staining solution according to the DIG Nucleic Acid Detection Kit protocol (Roche Diagnostic). We then performed light microscopic observation of serial sections of histologic specimens.

### Availability of supporting data

The nucleotide sequence(s) reported in this paper has been submitted to the GenBank/EMBL Data Bank with accession number(s) KP329574.

## Abbreviations

DGK: Diacylglycerol kinase
DG: Diacylglycerol
PA: Phosphatidic acid
PKC: Protein kinase C
RT-PCR: Reverse transcription-polymerase chain reaction
GAPDH: Glyceraldehyde-3-phosphate dehydrogenase
DIG: Digoxigenin
PBS: Phosphate buffered saline
Sg: Spermatogonium
Ps: Primary spermatocyte
Ss: Secondary spermatocyte
Rs: Round spermatid
Es: Elongated spermatid
Lc: Leydig cell
Pf: Primary follicle
Sf: Secondary follicle
Mf: Mature follicle
Cl: Corpus luteum
Gc: Granulosa cell
M: Medulla
Oe: Oviductal epithelium
L: Luminal epithelium
E: Endometrium
Ug: Uterine gland
